# The glutamate metabotropic receptor 5 (*GRM5*) gene is associated with beef cattle home range and movement tortuosity

**DOI:** 10.1186/s40104-022-00755-7

**Published:** 2022-09-15

**Authors:** Cristian A. Moreno García, Huitong Zhou, David Altimira, Robyn Dynes, Pablo Gregorini, Sadeepa Jayathunga, Thomas M. R. Maxwell, Jonathan Hickford

**Affiliations:** 1 Department of Agricultural Sciences, Faculty of Agriculture and Life Sciences, Lincoln University, PO Box 85084, Lincoln, 7647 New Zealand; 2 Exertion Games Lab, 8 Scenic Boulevard, Clayton, Victoria 3800 Australia; 3 AgResearch Limited, Lincoln Research Centre, Cnr Springs Road and Gerald Street, Lincoln, 7674 New Zealand; 4 Scion, Tītokorangi Drive (formerly Long Mile Rd), Rotorua, 3010 New Zealand

**Keywords:** Animal personality, Breeding programmes, Genetic associations, Grazing distribution, Grazing patterns, Steep and rugged terrain

## Abstract

**Background:**

The grazing behaviour of herbivores and their grazing personalities might in part be determined genetically, but there are few studies in beef cattle illustrating this. In this study, we investigated for first time the genetic variation within a candidate ‘grazing gene’, the glutamate metabotropic receptor 5 gene (*GRM5*), and tested associations between variation in that gene and variation in grazing personality behaviours (GP-behaviours) displayed by free-ranging cows during winter grazing in the steep and rugged rangelands of New Zealand. Mature beef cows (*n* = 303, from 3 to 10 years of age) were tracked with global positioning system (GPS) and, with 5-minutes (min) relocation frequency, various GP-behaviours were calculated. These included horizontal and vertical distances travelled, mean elevation, elevation range, elevation gain, slope, home range and movement tortuosity, variously calculated using daily relocation trajectories with repeated measurements (i.e., 7 to 24 days (d)) and satellite-derived digital elevation models (DEM). The different GP-behaviours were fitted into mixed models to ascertain their associations with variant sequences and genotypes of *GRM5*.

**Results:**

We discovered three *GRM5* variants (*A, B* and *C)* and identified the six possible genotypes in the cattle studied. The mixed models revealed that *A* was significantly associated with elevation range, home range and movement tortuosity. Similarly, *GRM5* genotypes were associated (*P* < 0.05) to home range and movement tortuosity, while trends suggesting association (*P* <  0.1) were also revealed for elevation range and horizontal distance travelled. Most GP-behaviour models were improved by correcting for cow age-class as a fixed factor. The analysis of GP-behaviours averaged per cow age-class suggests that grazing personality is fully established as beef cows reached 4 years of age. Home range and movement tortuosity were not only associated with *GRM5* variation, but also negatively correlated with each other (*r* = − 0.27, *P* < 0.001).

**Conclusions:**

There seems to be a genetically determined trade-off between home range and movement tortuosity that may be useful in beef cattle breeding programmes aiming to improve the grazing distribution and utilisation of steep and rugged rangelands.

**Supplementary Information:**

The online version contains supplementary material available at 10.1186/s40104-022-00755-7.

## Background

The selection and breeding of animals with desirable morphological and physiological characteristics has led to greater fitness and productivity in livestock farming systems. During the domestication of these animals and the establishment of livestock systems, behavioural characteristics have been equally or even more important than other production characteristics because of the close interaction with humans [1], yet measuring behaviour and objectively quantifying differences among individuals remains a challenge.

While the selection for animals with sets of behaviours suitable for safe handling and production has been practised along the animal domestication [1]; the realisation, conceptualisation and acceptance of animal personality is rather new [2]. This stated, similar (and probably) more accepted concepts such as temperament and behavioural syndromes [3, 4] have been around for longer. Regardless, we have limited knowledge of how individual personalities affect livestock production systems, the welfare of non-human animals, and the ecological functions and services associated with livestock production, such as the carbon cycle, nutrient redistribution and the quality of water.

The evidence shows that animal personality varies among individuals and that it affects livestock production and animal fitness [5, 6]. It can also be stated that to some extent, heritable factors determine behavioural characteristics and even personalities [7–9]. Thus, animal personality is becoming an important criterion in livestock breeding programmes [10, 11].

The concept of animal personality in foragers [12, 13] is rather novel. It is therefore not surprising that there are few reports describing genetic effects in the grazing personality of cattle [14], as well as a lack of candidate genes that might control such behaviours (but see [15, 16]). However, in one suggested model of grazing personalities (the GP-model [12]) it is postulated that distinctive grazing personalities might be determined by variations in ‘grazing-related genes’. What-is-more, these genes could be modulated by epigenetic mechanisms that control their activities through interactions with the social and biophysical environments, with this ultimately affecting the animal’s behaviour.

Genetic models are useful tools for the identification and study of candidate genes related to the physiology, behaviour and cognitive abilities. For example, Bakker and Oostra [17] studied the *Drosophila* model for fragile-X syndrome and found individuals with arrhythmic and erratic patterns of locomotor activities and abnormal circadian behaviour, which were regulated by the metabotropic glutamate receptor 5 protein (GRM5). Subsequently, Jew et al. [18] suggested that GRM5 controlled neural synaptic plasticity, and that in turn this modulated the locomotor reactivity of mice to novel environments. Jew’s et al. results showed direct association between GRM5 and locomotor reactivity to a novel environment, increased and decreased exploratory behaviour and, activity levels of mice. Subsequently, Wu et al. [19] reported that GRM5 in the forebrain GABAergic neurons of mice modulated locomotor activity, and in this way it affected their horizontal and vertical distances travelled and the time spent moving. These authors reported that mice with genotypic variation in the glutamate metabotropic receptor 5 gene (*GRM5*) displayed different levels of activity in familiar and unfamiliar environments.

The metabotropic glutamate receptors (GRMs) are G-protein coupled receptors that have been categorised into three groups according to their sequence similarity and intracellular signalling mechanisms. The GRMs 1 (GRM1) and 5 (GRM5) are members of receptor group 1, which couple with phospholipase C and have similar functions or effects. Bossi et al. [20] reported GRM5 interactions with GRM1 that affected the motor coordination of mutant mice, and in a recent study, Gray et al. [21] concluded that the stimulation of GRMs Group 1 increased the activity of Cav2.3 R-type voltage gated Ca^2+^-channels in hippocampal neurons. This led to hyperactivity at the neural synapses and aberrant calcium spiking in both male and female, and it caused deficient short-term memory, increased activity, and increased exploratory behaviour.

Earlier work with *GRM5*-knockout mice also suggested effects related to spatial cognitive ability. For example, Lu et al. [22] observed impairment in the acquisition and use of spatial information and persistent strengthening of neural synapses (i.e., long-term potentiation). Such results were consistent with Bliss and Collingridge’s findings [23], which linked neural potentiation with memory and spatial learning. Taken together, the literature would suggest that GRM5 may either directly or indirectly control animal activity and cognitive behaviours related to the exploration and use of space.

In 2015, Bailey et al. [15] conducted a study seeking genetic associations with the grazing behaviour of beef cows recorded using GPS-tracking collars on five farms in the United States of America (USA). The cattle were screened to identify quantitative trait loci (QTL) related to terrain-use indices. Two of the QTLs overlapped *GRM5* on bovine chromosome 29, and these explained 18% and 24% of the total variation in the study’s so-called ‘rough’ grazing index. The reported associations between a QTL overlapping *GRM5* and the rough index made *GRM5* a candidate gene to explain the phenotypic variation in GP-behaviours of beef cattle.

Accordingly, for this study, we hypothesize that nucleotide sequence variation in bovine *GRM5* may be associated with behaviours that underpin the grazing personalities displayed by beef cattle, and hence research was undertaken to ascertain whether genetic variation exists in *GRM5*, and if it existed, to explore its association with grazing personality behaviours in beef cattle.

## Methods

### Cattle investigated and phenotypic data collection

The cows studied (*n* = 306) ranged in age from 3 to 10 years, and they were categorised into three age groups: ‘class 1’ (under four years of age), ‘class 2’ (four to five years of age) and ‘class 3’ (six or more years of age). They were either Hereford cattle (*n* = 224) or Angus × Hereford cross cattle (*n* = 82), with the crosses present on two farms. For the Hereford cattle, most of the cows were from registered studs, and pedigree information (i.e., sire and dam identities) could be gathered from publicly available sources [24], or directly from the farmer. For cows with unknown pedigree (*n* = 82), three ‘notional sire’ identities were allocated in three of the fourteen mobs (i.e., cattle groups between and within farms and sampling years) to avoid redundancy among herds (i.e., cattle groups within a given farm), farms (*n* = 4) or years (*n* = 2).

For the 2019 sampling, fifteen cows were selected within the existing breeding herds at four farms and GPS-tracked. This was undertaken with modified tracking collars that contained i-gotU GT-600 GPS data loggers (Mobile Action Technology Incorporated, Taipei, Taiwan) and additional rechargeable batteries to prolong running time in the field. Subsequently, in 2020, ninety cows were selected within a single herd from each farm for GPS-tracking and the tracking collar deployments were carried out, one farm after the other, during the targeted grazing season. Due to failure of some GPS devices, several deployments did not yield usable data (see details below).

Grazing behaviour was recorded in steep and rugged rangelands of Canterbury, New Zealand, over the autumn and winter period (approximately between April and August). As is commonly practised on New Zealand commercial farms, mated cows were moved to graze higher rangelands immediately after weaning (in April). They remained in these uplands until commencement of the calving season in spring (August–September), and were grazed in a ‘free-range’ system, on the relatively large (average size 34.5 ha) and uncultivable paddocks of the so-called ‘New Zealand hill country’ (see Tozer et al. [25] for terrain description). The data set for statistical analysis comprised 303 cows (except for results in Table 3) from four farms, sampled over two years that sum up to fourteen mobs (i.e., different herds within and between farms and years) from 73 sires and five *GRM5* genotypes (genotype *AA* excluded).

For each collar deployment, individual cow trajectories for the duration of the grazing period were created using the R package ‘adehabitatLT’, which contains functions capable of dealing with the analysis of animal movement [26]. In these analyses, a combination of turning angle and speed between geolocations was used to identify GPS outliers [27], and then the trajectories excluding the outliers were recalculated. The shuttle radar topography mission digital elevation model (DEM) raster of New Zealand (16 m resolution) was downloaded from Land Information New Zealand [28] and additional rasters were created for slope and aspect using the 3D Analyst toolbox of ArcMap™ [29]. The annotation with data of elevation, slope and aspect for each GPS data point was obtained by extracting values using the R package ‘raster’ [30].

With assistance from the R package ‘dplyr’ [31], a number of behaviours describing grazing personalities (GP-behaviours) were calculated for each cow. First, they were calculated on a daily basis, but days with a recording rate under 75% (i.e., less than 216 data points recorded out of 286, for locations recorded at a 5-min intervals) were not included. Next, the mean of each GP-behaviour was calculated across the days for each cow. The GP-behaviours included: the daily horizontal distance travelled, the daily vertical distance travelled, the daily three-dimensional distance travelled, the daily elevation range, the daily elevation gain, the relative elevation mean, the relative elevation 85^th^ quantile, the relative elevation range, the daily slope 85^th^ quantile, the daily home range (using the minimum convex polygon method) and the daily movement tortuosity (using the spatial search pattern [13, 32]). See Table 1 for a detailed descriptions of GP-behaviours.

**Table 1:** List of grazing personality behaviours with abbreviations, units, data transformations and description of calculations

	Abbreviation	Units	Transformation	Description
Daily horizontal distance travelled	ho_dist	m/d	Log transformed	Distance calculated as the sum of distances between consecutive GPS^a^ data points per d using two dimensions (i.e. Easting and Northing) of the UTM^b^ projection
Daily vertical distance travelled	ve_dist	m/d	Log transformed	Distance calculated as the sum of the absolute difference in elevation between consecutive GPS data points per d using a DEM^c^
Daily three-dimensional distance travelled	3D_dist	m/d	Log transformed	Distance calculated as the sum of distances between GPS data points per d using three dimensions (i.e. Eastern and Northing of UTM and elevation difference from DEM)
Daily elevation range	ele_range	m	Log transformed	Range of elevation computed as calculated as the difference between the daily maximum and minimum elevation
Daily elevation gain	ele_gain	m/d	Log transformed	Sum of positive changes of elevation between consecutive GPS data points as depicted from a DEM
Relative elevation mean	rel_ele	0–1 scale		In any given day, ratio between the cows’ mean elevation minus the minimum elevation of the herd and the elevation range of the herd
Relative elevation 85^th^ quantile	rel_ele85	0–1 scale		In any given day, ratio between the cows’ 85^th ^quantile of the elevation minus the minimum elevation of the herd and the elevation range of the herd
Relative elevation range	rel_ele_range	0–1 scale		In any given day, ratio between the cows’ elevation range and the elevation range of the herd
Daily slope 85^th^ quantile	slope85	Percentage		85^th^ quantile of the slope across GPS data points per day as depicted from a DEM
Daily home range	hr_mcp	ha/d	Log transformed	Explored area estimated by calculating the minimum convex polygon depicted from all GPS data points per day using the R package ‘*adehabitatHR*’
Daily searching pattern tortuosity	sp_tortuosity	m/ha	Log transformed	Movement tortuosity estimated as the ratio between daily horizontal distance and daily home range

^a^*GPS* Global Positioning System fixes recorded with iGot-U GT600, Mobile Action

^b^*UTM* Universal Transverse Mercator

^c^*DEM* Digital Elevation Model with an 16 m × 16 m spatial resolution

A minimum of 7 days (d) of GPS tracking data were deemed sufficient to represent consistent grazing behaviours, thus any cow with six or fewer days of data collection was excluded from the study. For each cow, the first 7 to 28 daily trajectories recorded were analysed from the start of GPS deployment, when herds grazed in rolling or steeper rangeland terrain (i.e., when the median daily slope for the relocations of the herd was greater than eight angular degrees (°)) based on slope classes for New Zealand [33]. Overall, GP-behaviours for 303 cows were analysed.

### Blood sampling and polymerase chain reaction-single strand conformation polymorphism (PCR-SSCP) analysis of *GRM5*

Individual blood samples from the nicked ears of the cattle were collected onto TFN paper (Munktell Filter AB, Sweden), and genomic DNA used for polymerase chain reaction (PCR) amplification was purified from the dried blood spot using a two-step procedure described by Zhou et al. [34].

Human *GRM5* was first described as having nine coding exons, with lengths ranging from 96 to 940 base pairs (bp) [35]. Based on this, a human *GRM5*–202 (ENST00000305447.5) sequence was analysed to ascertain which region of bovine *GRM5* may be suitable for further molecular analysis. Exon V (247 bp) (hereafter referred to as exon 5) was chosen for analysis, as this exon encodes part of the receptor-binding region [35] and has more sequence variation described in Ensembl (ENSBTAG00000048061) than other regions of the gene.

A pair of PCR primers were then designed to amplify the *GRM5* exon 5 region based on the sequence ENSBTAG00000048061. These primer sequences were 5′-AGAATCCATAAAGAGCTACAG-3′ and 5′-GATCAGGCTCTGGTGTCTAG-3′, and the primers were synthesised by Integrated DNA Technologies (Coralville, IA, USA).

The PCR amplifications with these primers were performed in a 15-μL reaction. These contained the DNA of one punch of TFN paper, 150 μmol of each deoxyribonucleoside (dNTP) (Bioline, London, United Kingdom), 0.25 μmol of each primer, 0.5 U of Taq DNA polymerase (Qiagen, Hilden, Germany), 2.5 mmol Mg^2+^, 1× reaction buffer supplied with the enzyme and distilled water to make up volume. The thermal profile for amplification consisted of 2 min at 94 Celsius degrees (°C), followed by 35 cycles of 30 seconds (s) at 94 °C, 30 s at 60 °C, and 30 s at 72 °C, with a final extension of 5 min at 72 °C.

The PCR amplicons were screened for sequence variation using single strand conformation polymorphism (SSCP) analysis. Each amplicon (0.7 μL) was mixed with 7 μL of loading dye (98% formamide, 10 mmol EDTA, 0.025% bromophenol blue, and 0.025% xylene cyanol). After denaturation at 95 °C for 5 min, the samples were rapidly cooled on wet ice and then, electrophoresed in 16 cm × 18 cm, 14% acrylamide:bisacrylamide (37.5:1) (Bio-Rad) gels in 0.5× TBE buffer at 6 °C and 370 Volts (V) for 19 hours (h). The gels were silver-stained according to the method of Byun et al. [36].

### Sequencing of variants and sequence analysis

PCR amplicons representing different SSCP banding patterns from cattle that appeared to be homozygous were sequenced in both directions using Sanger sequencing at the Lincoln University DNA Sequencing Facility (Lincoln University, Canterbury, New Zealand). Nucleotide sequence alignments and translation to amino acid sequences were undertaken using DNAMAN (version 5.2.10, Lynnon BioSoft, Vaudreuil, QC, Canada).

### Statistical analyses

Statistical analyses were conducted using R [37]. For data aggregated at the daily level, skewness, kurtosis and normality were graphically evaluated by plotting histograms with their corresponding theoretical normal distribution curves and with Q-Q plots. When needed, logarithmic transformation was utilised to better fit the data into a normal distribution. Overall, the dataset comprised 6142 daily-aggregated observations.

For data aggregated at the cow level (i.e., averaged across 7–28 d of records), Pearson correlation coefficients were calculated between the eleven GP-behaviours using ‘rcorr()’ from the R package ‘Hmisc’ [38]. The correlations were calculated based on data from the 303 cows (three cows with *GRM5* genotype *AA* excluded from analysis), except for home range and movement tortuosity (*n* = 299) because of missing values.

Linear mixed models (LMM) and generalised linear mixed models (GLMM) were fitted to the GP-behaviours to assess their associations with *GRM5* variants and genotypes using the R package ‘lme4’ [39]. The LMMs were fitted to GP-behaviours with a Gaussian distribution (e.g., daily horizontal distance traveled), whereas the GLMMs were used with bounded GP-behaviours (i.e., those scaled between 0 and 1, e.g., relative elevation mean) set with binomial distributions, which apply a logit transformation. The GLMM-binomial distribution was preferred over using beta distribution [40], in order to correct for random factors.

Unbalanced repeated measurements were nested into a cow identity factor. The effects of farm, sampling year, mob, sire and genotype were tested as potential random explanatory factors. Breed effect (i.e., Hereford vs. Angus × Hereford cross) was not independently assessed because the number of Angus × Hereford cattle was small (*n* = 82) and some of the genotypes were rare, and whilst breed might be affecting the various phenotypic measures the cattle studied cannot be claimed to be representative of the breed as a whole. There were 29 half-sister cows that shared the same sire but were part of different mobs.

For each GP-behaviour, the random and fixed explanatory factors were selected in two steps. First twelve models were run with several combinations of random factors only (i.e., cow identity, farm, sampling year, mob, and *GRM5* genotype). The model with the best compromise of statistics (i.e., least degrees of freedom, lowest Akaike information criterion (AIC) [41], lowest Bayesian information criterion (BIC) and lowest factor significance evaluated using the ‘anova()’ function), was selected for further evaluation. In a second step, the random factor selected models were then fitted with the corresponding fixed factors, i.e., presence/absence of the variants or *GRM5* genotype (the predictor variables under evaluation), to create variant and genotype models respectively, and with cow age-class. Using the same criteria as above to reach the best compromise of AIC, BIC and ANOVA, a final model with or without the cow age-class factor was fitted.

To carry out the comparison of models, the maximum likelihood method was used. Once the random and fixed factors were set, models were fitted using restricted maximum likelihood procedures [42]. The suitability of the selected models was assessed with plots of residual versus fitted data, and with the criteria of accepting models with up to 5% of scaled residuals beyond the ±3 limits. Associations of fixed factors (variants, genotypes and cow age-classes) were assessed with the Satterthwaite’s method using the R package ‘emmeans’ [43], and *post-hoc* analyses (pairwise comparisons) were undertaken using the Benjamini-Hochberg method [44, 45] in ‘emmeans’. Groups that were different at *P* < 0.05 were labelled with different letters using the ‘multcompView’ package in R [46].

Dominance models were fitted by testing the effect of the presence/absence of variants and genotype models were fitted with the identified genotypes. Cows with low genotype frequency in the cattle studied (i.e., 5%) were excluded from the various statistical analyses.

## Results

Eleven grazing personality behaviours were derived by combining GPS DEM-annotated data from free-ranging cows grazing rangeland. These were calculated on a daily basis, and subsequently averaged across 7–28 d for each individual cow.

### Correlation of grazing personality behaviours

The Pearson correlation coefficients (*r*) between the grazing personality behaviours are summarised in Table 2.

**Table 2:** Pearson correlation coefficients for the grazing personality behaviours (GP-behaviours)

GP-behaviours^a^	ho_dist	ve_dist	3D_dist	ele_range	ele_gain	rel_ele	rel_ele85	rel_ele_range	slope85	hr_mcp
ve_dist	0.70^***b^									
3D_dist	1.00^***^	0.72^***^								
ele_range	0.07	0.41^***^	0.08							
ele_gain	0.70^***^	1.00^***^	0.72^***^	0.41^***^						
rel_ele	−0.13^*^	0.19^**^	0.00^*^	0.68^***^	0.19^**^					
rel_ele85	−0.14^*^	0.11^*^	−0.13^*^	0.71^***^	0.11	0.97^***^				
rel_ele_range	0.41^***^	0.12^*^	0.40^***^	0.28^***^	0.12^*^	0.18^**^	0.36^***^			
slope85	−0.26^***^	0.38^***^	−0.23^***^	0.60^***^	0.38^***^	0.43^***^	0.42^***^	−0.09		
hr_mcp	0.32^***^	−0.13^*^	0.30^***^	−0.09	−0.12^*^	−0.24^***^	−0.08	0.54^***^	−0.27^***^	
sp_tortuosity	0.48^***^	0.56^***^	0.49^***^	−0.07	0.55^***^	−0.03	−0.15^*^	−0.17^**^	−0.04	−0.27^***^

^a^Daily horizontal distance travelled (ho_dist), daily vertical distance travelled (ve_dist), daily three-dimensional distance travelled (3D_dist), daily elevation range (ele_range), daily elevation gain (ele_gain), relative elevation mean (rel_ele), relative 85 quantile of elevation (rel_ele85), relative elevation range (rel_ele_range), 85 quantile of daily slope (slope85), daily home range (hr_mcp) and daily movement tortuosity (sp_tortuosity)

^b^Bolded values indicate moderate (*r* = 0.3–0.5) and strong (*r* > 0.5) correlations

*P < 0.05, **P < 0.01, ***P < 0.001

Daily horizontal distance travelled, daily three-dimensional distance travelled, daily vertical distance travelled and daily elevation gain were highly positively correlated with each other, and they had positive correlations that ranged between *r* = 0.48 and *r* = 0.56 with daily movement tortuosity. Moderate positive correlations were found between the daily horizontal distance travelled and the daily home range (*r* = 0.32) and the daily three-dimensional distance travelled and the daily home range (*r* = 0.30). The daily horizontal distance travelled was moderately positively correlated with relative elevation range (*r* = 0.41) and the daily three-dimensional distance travelled was moderately positively correlated with relative elevation range (*r* = 0.40). The daily vertical distance travelled had a moderate positive correlation (*r* = 0.38) with the 85^th^ quantile of daily slope, and the daily elevation range and daily elevation gain were also positively correlated with the 85^th^ quantile of daily slope (*r* = 0.60 and *r* = 0.38 respectively).

When looking at relative GP-behaviours (i.e., those calculated by comparing the individual behaviour with the mean behaviour displayed by animals of the same herd), strong positive correlations were revealed between daily elevation range and relative elevation mean (*r* = 0.68), as well as with the 85^th^ quantile of relative elevation (*r* = 0.71). Similarly, the 85^th^ quantile of daily slope correlated positively (*r* = 0.43 and *r* = 0.42 respectively) with relative elevation mean and the 85^th^ quantile of elevation. Finally, the daily home range was strongly correlated (*r* = 0.54) with the relative elevation range.

### Genetic variation in *GRM5* exon 5

**Fig. 1:**
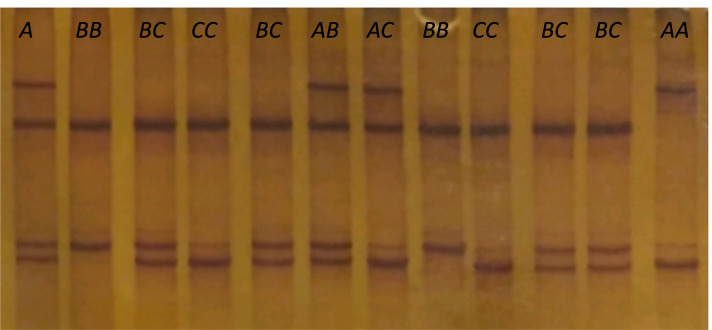
Banding patterns of *GRM5* genotypes. Banding patterns of various genotypes of the bovine glutamate metabotropic receptor 5 gene (*GRM5*) exon 5 region obtained from polymerase chain reaction-single strand conformation polymorphism (PCR-SSCP) analyses

The nucleotide sequence variation in exon 5 of *GRM5* was investigated in 306 adult cows, albeit only 303 of these were subject to further statistical analyses. After PCR-SSCP analyses of the *GRM5* exon 5 fragment, three distinctive banding patterns corresponding to homozigous variants named *A, B* and *C* were identified. Figure 1 shows the three homozygous and several heterozygous banding patterns.

DNA sequencing of the PCR products of these three variants revealed three new and different nucleotide sequences (GenBank accessions numbers OK078019, OK078020 and OK078021) with two previously reported nucleotide substitutions (rs43744222 and rs210610001). These substitutions, if expressed, would not change the amino acid sequence.

The six possible genotypes of the three variants were all identified (Table 3). Genotype *AA* was the least common, and across the four farms was present in only 1% (*n* = 3) of the cows. Genotypes *BC* and *CC* were the most common, with frequencies of 36% and 35% respectively, and together these genotypes accounted for 65–79% of cows on any given farm.

**Table 3:** Number of cows (and percentage frequency) per farm of each glutamate metabotropic receptor 5 gene (*GRM5*) genotype of four beef farms in the Canterbury Region of New Zealand

Genotype	Farm				
	1	2	3	4	Total
*AA*	0 (0%)	1 (2%)	1 (1%)	1 (1%)	3 (1%)
*AB*	4 (5%)	3 (5%)	4 (5%)	7 (9%)	18 (6%)
*AC*	11 (13%)	6 (10%)	5 (6%)	9 (12%)	31 (10%)
*BB*	5 (6%)	3 (5%)	20 (24%)	10 (13%)	38 (12%)
*BC*	36 (43%)	23 (38%)	25 (30%)	25 (32%)	109 (36%)
*CC*	27 (33%)	25 (41%)	29 (35%)	26 (33%)	107 (35%)
Total	83	61	84	78	306

### Selecting random explanatory factors for the linear mixed models

For each GP-behaviour, twelve different combinations of random factors were assessed, and the best combination of the lowest AIC (Table S1) and BIC (not shown), lowest number of degrees of freedom (Table S1) and those that were statistically significant by an ANOVA comparison of models (results not shown) was selected. The selected combination of random factors for each GP-behaviour are indicated with bolded and underlined AIC values (Table S1). The models for horizontal distance travelled and three-dimensional distance travelled were ‘best’ corrected using sampling year as a random factor (i.e., 2019 or 2020) and within each year of the farm (farm: sampling year) (RF4 in Table S1). Models for vertical distance travelled, relative elevation, 85^th^ quantile of relative elevation and 85^th^ quantile of slope were ‘best’ corrected with the factor mob, and for the remaining five GP-behaviours a combination of mob and sire was the selected random factor correction.

Nearly 75% of the cows tracked in this study were Herefords and the rest Angus × Hereford crosses mostly from farm 2 (16%). Thus, farm effects could have been confounded with breed effects if the crossbred cows actually differed from the purebred cows. However, since purebred cows dominated the dataset, differences in breed are considered small in our dataset (in comparison to other factors such as the farm effect) and therefore negligible for the correction of models. The models were therefore corrected for farm effects only and breed was not included as factor. As such, the results chiefly represent variability within Hereford cattle.

Selected genotype models were assessed by plotting residuals versus fitted values. All models had less than 1% of the scaled residuals exceeding the limits and the residuals were mostly randomly distributed (Figs. S1 and S2).

### Cow age-class as a fixed explanatory factor

Tables 4 and S2 reveal summaries for correcting the *GRM5* variant and genotype models respectively with cow age-class as a fixed explanatory factor. Out of eleven variant and genotype models and for the same GP-behaviours, seven were improved by correcting for cow age-class: horizontal and 3D distances, relative elevation, 85^th^ quantile of relative elevation, 85^th^ quantile of daily slope, home range and movement tortuosity.

**Table 4:** Associations between bovine glutamate metabotropic receptor 5 gene (*GRM5*) variants and grazing personality behaviours (GP-behaviours)

GP-behaviour^**a**^	Cow age-class (***P***-value)^**b**^	Variant	Marginal mean^**c**^ (standard error)	***P***-value^**d**^
ho_dist, m/d	**0.038**	*A*	3837 (± 1120)	0.847
	**0.039**	*B*	3843 (±1120)	0.741
	**0.037**	*C*	3874 (± 1130)	0.213
ve_dist, m/d	0.697	*A*	566 (±76)	0.461
	0.701	*B*	557 (±74)	0.638
	0.703	*C*	555 (±73)	0.916
3D_dist, m/d	**0.044**	*A*	3900 (± 1141)	0.870
	**0.044**	*B*	3903 (± 1139)	0.749
	**0.043**	*C*	3935 (± 1149)	0.223
ele_range, m	0.413	*A*	71.0 (±9.0)	**0.008**
	0.407	*B*	66.3 (±8.4)	0.833
	0.402	*C*	66.3 (±8.4)	0.729
ele_gain, m/d	0.697	*A*	283 (±38)	0.452
	0.699	*B*	279 (±37)	0.485
	0.704	*C*	277 (±37)	0.963
rel_ele (0–1)	**0.018**	*A*	0.42 (±0.06)	0.212
	**0.017**	*B*	0.44 (±0.05)	0.263
	**0.018**	*C*	0.46 (±0.05)	0.556
rel_ele85 (0–1)	**0.006**	*A*	0.67 (± 0.05)	0.934
	**0.006**	*B*	0.67 (±0.05)	0.274
	**0.006**	*C*	0.68 (±0.05)	0.749
rel_ele_range (0–1)	0.286	*A*	0.51 (±0.09)	0.354
	0.266	*B*	0.50 (±0.09)	0.610
	0.277	*C*	0.50 (±0.09)	0.971
slope85 (0–1)	**0.023**	*A*	0.47 (±0.08)	0.138
	**0.026**	*B*	0.44 (±0.08)	0.848
	**0.025**	*C*	0.43 (±0.08)	0.205
hr_mcp, ha/d)	**0.008**	*A*	7.86 (±0.83)	**0.021**
	**0.008**	*B*	7.22 (±0.74)	0.285
	**0.008**	*C*	7.39 (±0.75)	0.273
sp_tortuosity, m/ha	**0.006**	*A*	556 (±74)	**0.003**
	**0.006**	*B*	611 (±79)	0.139
	**0.007**	*C*	598 (±77)	0.476

^a^See GP-behaviours abbreviations and details in Table 1

^b^Significance level of ANOVA test for comparison of models with and without cow age-class as fixed factor. Bold values indicate significance (*P* < 0.05)

^c^Marginal mean for variant presence in measured units (back-transformed from the log scale as required) as estimated with linear mixed models

^d^Significance level for Satterthwaite’s method *t*-test of presence/absence of variant. Bold values indicate significance (*P* < 0.05)

For any given GP-behaviour, the three *GRM5* variant models showed similar results, with an explanatory factor correcting all three models, or none of them. *P*-values ranged between 0.01 and 0.05 for horizontal and 3D-distance travelled, relative mean elevation, relative 85^th^ quantile elevation and 85^th^ quantile slope (Table 4). For home range and movement tortuosity, *P*-values were below 0.01 (Table 4). The significance level for each GP-behaviour was similar for the variant and the genotype models.

### Association of *GRM5* variants and genotypes with grazing personality behaviours

Using linear mixed models, associations between the presence/absence of the *GRM5* variants and GP-behaviours were investigated. The presence of variant *A* was associated with elevation range, home range and movement tortuosity (Table 4). Trends to association (*P* < 0.2) were found for variant *A* and the 85^th^ quantile of slope (*P* = 0.138) and for variant *B* with movement tortuosity (*P* = 0.136). No associations were detected with variant *C*.

The *GRM5* genotype models (Table S2) revealed associations with home range and movement tortuosity (see below genotypes *post-hoc* analysis). Trends suggesting association (*P* < 0.1) were found between the *GRM5* genotypes and elevation range, as well as with the horizontal distance travelled and three-dimensional distance travelled. Overall, this suggested a high degree of consistency between the variant presence/absence models and the genotype models.

The effect of the fixed factors was consistent in the variant and genotype models. Thus, if cow age-class had an effect in the variant presence/absence models, this effect was observed in the corresponding genotype model. Similarly, when genetic associations were revealed in the variant models, such genetic effects were reflected in the genotype models too.

### *Post-hoc* comparisons of *GRM5* genotypes and cow age-class

The *GRM5* genotypes associations with home range and movement tortuosity (Table S2) were related to cow age-class. For these two GP-behaviours, an analysis of the main effect of genotype across cow age-class and *post-hoc* analyses were conducted. Results of *post-hoc* analyses revealed significant differences in the combined effects of *GRM5* genotypes and cow age-classes for both GP-behaviours (Table 5).

**Table 5:** *Post-hoc* comparisons between groups of glutamate metabotropic receptor 5 gene (*GRM5*) genotypes and cow age-classes that had significant association with grazing personality behaviours (GP-behaviours)

***GRM5*** genotype	Cow age-class	Marginal mean^**a**^ (standard error)	Degrees of freedom	BH^**b**^ (*P* < 0.05)
Home range, ha/d				
* AB*	1	9.74 (±1.52)	62	a
* AC*		9.67 (±1.48)	55	a
* BB*		8.16 (±1.26)	57	bcd
* BC*		8.96 (±1.32)	50	ab
* CC*		9.01 (±1.34)	51	ab
* AB*	2	7.43 (±0.83)	23	abc
* AC*		7.37 (±0.77)	18	ab
* BB*		6.22 (±0.65)	18	de
* BC*		6.83 (±0.67)	14	bcd
* CC*		6.87 (±0.68)	14	bcd
* AB*	3	6.77 (±0.80)	29	bcd
* AC*		6.72 (±0.76)	24	bcd
* BB*		5.67 (±0.64)	24	e
* BC*		6.23 (±0.66)	20	cde
* CC*		6.26 (±0.67)	20	cde
Searching pattern tortuosity, m/ha				
* AB*	1	434 (±73)	35	j
* AC*		456 (±75)	33	ij
* BB*		534 (±89)	34	cdefgh
* BC*		495 (±80)	31	ghi
* CC*		484 (±79)	31	hij
* AB*	2	595 (±81)	17	efghi
* AC*		626 (±83)	15	cdefgh
* BB*		732 (±97)	15	ab
* BC*		679 (±88)	14	abcd
* CC*		663 (±86)	14	abcdef
* AB*	3	610 (±86)	19	dfghi
* AC*		641 (±88)	17	bcdefg
* BB*		751 (±103)	17	a
* BC*		696 (±93)	16	abce
* CC*		680 (±91)	16	abcdef

^a^Response marginal mean in measured units (back-transformed from the log scale as needed)

^b^Different letters indicate significantly different groups calculated with a pairwise comparison using the Benjamini-Hochberg method (*P* < 0.05)

#### Home range

Here is presented the selected linear mixed model for home range (‘lme4’ notation):

- Response variable: log (hr_mcp)
- Fixed factors: GRM5_genotype + cow_age_class +
- Random factors: (1|cow_id) + (1|mob_id) + (1|sire_id)

Where hr_mcp refers to home range and id denotes the identifier.

The main effects of cow age and genotype were assessed with a mean comparison using the Benjamini-Hochberg method for the adjustment of *P*-values. When the daily home range by cow age (i.e., home ranges averaged over genotype levels) was analysed, cows younger than 4 years of age (class 1) explored larger areas (9.09 ha per day (ha/d)) than cows of 4–5 years of age (class 2; 6.93 ha/d; *P* = 0.039) and older cows of six or more years of age (class 3; 6.32 ha/d; *P* = 0.014). The daily home range explored by the 4–5 years old cows was also larger than that of the older cows (*P* = 0.046). The main effects of genotype in daily home range across cow age-classes (i.e., home ranges averaged over levels of cow age-class) was not significant (*P* < 0.05), but it tended to decrease, with *AB* and *AC* being greater than *CC* and *BC*, which were greater than *BB* (7.88 ha/d, 7.82 ha/d, 7.29 ha/d, 7.25 ha/d and 6.60 ha/d, respectively).

The results of the combined effects of genotype and cow age revealed that the daily home range of an individual cow calculated with the minimum convex polygon method ranged between 5.67 ha/d (genotype *BB*, cow age-class 3) and 9.74 ha/d (genotype *AB*, cow age-class 1) (Table 5). Cows with genotype *BB* displayed among the lowest daily home ranges in age-classes 2 and 3, but for the cows under four years of age (class 1), genotype *BB* cows had similar daily home ranges to the *BC* and *CC* genotype cows, but significantly smaller daily home ranges than the *AB* (*P* = 0.047) and *AC* cows (*P* = 0.032). Young cows (class 1) with genotypes *AB* and *AC* had larger daily home ranges than cows of any other genotype of age-classes 2 and 3; and within cow age-class 1, the daily home ranges of the *AB* and *AC* cows were larger than *BB* cows (see above). For age-classes 1, 2 and 3, cows of genotype *AC* displayed slightly smaller, but statistically similar daily home ranges to *AB* cows (*P* = 0.933, *P* = 0.933 and *P* = 0.933, respectively). Cows with genotypes *BC* and *CC* had intermediate values for their daily home ranges.

#### Movement tortuosity

Here is presented the selected linear mixed model for movement tortuosity (‘lme4’ notation):

- Response variable: log (sp-tortuosity)
- Fixed factors: GRM5_genotype + cow_age_class +
- Random factors: (1|cow_id) + (1|mob_id)

Where sp_tortuosity refers to movement tortuosity and id denotes the identifier.

Analysis of the main effects of genotype and cow age on daily movement tortuosity, revealed that cows in class 3 (six-years and older) displayed similar tortuous trajectories (674 m per hectare (m/ha)) than the middle-aged cows (class 2; 657 m/ha; *P* = 0.517). Younger cows of class 1 (less than four-years of age) had less tortuous trajectories (479 m/ha) than cows in both of the older age-classes (*P* = 0.005). The genotype main effects averaged by cow age-class revealed that cows with the genotype *BB* had the most tortuous trajectories (665 m/ha). This was followed by cows with genotypes *BC* and *CC* (616 m/ha; not significant *P* = 0.123, and 602 m/ha; a trend at *P* = 0.061, respectively). Cows with the genotypes *AC* (578 m/ha) and *AB* (540 m/ha) had straighter trajectories than *BB* genotypes (*P* = 0.021 and *P* = 0.012, respectively). Differences in movement tortuosity between cows of *AC* and *AB* genotype were trending towards a difference (*P* = 0.061).

When accounting for the combined effects of genotype and age, daily movement tortuosity estimated with the spatial search pattern ranged from 434 m/ha (genotype *AB*, age-class 1) to 751 m/ha (genotype *BB*, age-class 3) (Table 5). Cows of genotype *BB* and age-classes 2 and 3 displayed among the largest daily movement tortuosity, but having the *BB* genotype did not lead to much distinction in the young cows (age-class 1). In turn, young *BB* cows displayed similar movement tortuosity to other genotypes in age-classes 2 and 3, as well as to cows with genotypes *BC* and *CC* in age-class 1.

#### Cow age-class as main factor

Results presented above showed some of the differences among age-classes when computed as main factor. For example, home range significantly (*P* < 0.05) decreased from cow age-class 1 (9.09 ha/d) to classes 2 (6.93 ha/d) and 3 (6.32 ha/d). Similarly, movement tortuosity was significantly (*P* < 0.01) smaller for cows age-class 1 (479 m/ha) than both older classes suggesting that trajectories of the youngest class was straighter. Differences on movement tortuosity were not significant between cows of age-classes 2 (657 m/ha) and 3 (674 m/ha), albeit values tended to increase. This lack of difference might be largely explained by the significantly (*P* < 0.05) shorter horizontal distance travelled by cows in class 3 (3689 m/d) compared to cows in class 2 (3941 m/d) and, in lesser extent, by their difference in home range. On the contrary, the slope 85^th^ quantile significantly (*P* < 0.01) increased from the youngest cow age-class 1 having 37.5% (17 °) to age-class 2 with 45.8% (21 °) while even steeper 85^th^ quantile slope was recorded in cows of age-class 3, which reached 52.9% (24 °).

## Discussion

### Sets of correlated grazing personality behaviours

Senft et al. [47] provided clues about which grazing personality behaviours were relevant to describe grazing patterns and predict the distribution of cattle. Accordingly, eleven behaviours related to the grazing personality of beef cattle were measured or calculated in this study. In some cases, these behaviours were correlated and provide insights into behavioural trade-offs that could be affected by genetics. In other cases, the correlations between behaviours might be explained with other reasons or factors, and might not have their roots in behaviour or animal personality.

In the following paragraphs, we discuss in detail some examples of such correlations. We report correlations that do suggest behavioural trade-offs and even concatenated behaviours that ultimately might resemble differences in grazing personality. For example, cows that travelled longer distances on a daily basis (i.e., both horizontal distance travelled and three-dimensional distance travelled) had increased daily elevation gains and displayed more tortuous trajectories.

Longer distances travelled (i.e., horizontal distance travelled and three-dimensional distance travelled) were moderately and positively correlated with daily movement tortuosity (*r* = 0.48 and *r* = 0.49, respectively in Table 2), while daily vertical distance travelled had an even stronger correlation (*r* = 0.56) with daily movement tortuosity, meaning more twisted trajectories when elevation gains (and losses) increased. It was also found that cows that travelled longer distances (i.e., horizontal distance travelled and three-dimensional distance travelled), typically had larger daily home ranges (*r* = 0.32 and *r* = 0.30, respectively, in Table 2). In contrast, cows with greater vertical distance travelled (i.e., the sum of elevation gain and loss), typically explored smaller home ranges (*r* = − 0.13 in Table 2). Despite this, the daily horizontal and vertical distances travelled were positively correlated, and increased in both of these behaviours with larger or smaller home ranges, respectively. This suggests a trade-off between the size of the area explored and the extent of elevation change. Similarly, the negative correlation (*r* = − 0.27 in Table 2) between the daily home range and daily movement tortuosity suggests another trade-off, where the smaller the area explored, the more crooked the trajectories.

Browning et al. [32] analysed the grazing behaviour of mature Angus × Hereford cross cows grazing in the northern Chihuahuan Desert (NM, USA). They reported that as pasture regrew, the movement tortuosity estimated during grazing activity periods (75.3 m/ha) tended to increase (*r* = 0.62), while home range decreased (*r* = − 0.38). These results agree with our findings and further support the existence of a trade-off between the extent of the home range and the nature of the movement tortuosity. It does however need to be noted that there was a marked difference between the land being grazed in the two studies. Browning’s et al. [32] experiment was set up on flat desert land with a mean distance travelled of 6100 m/d and explored areas of 81.1 ha/d, versus our study, which was conducted in steep and rugged terrain where the average distance travelled was 3700 m/d and cattle explored 12.77 ha/d. Overall, the study undertaken here, revealed an average daily home range of nearly 13 ha/d, with a notably higher movement tortuosity of 629 m/ha, eight-fold larger than the value of 75.3 m/ha reported in Browning et al. [32]. Another difference between these studies is that Browning’s et al. [32] measurements accounted exclusively for grazing time, while our study used the total daily movement regardless of activity, (i.e., it was not just time spent grazing). Additionally, given the difference in latitude of these two studies, the effect of day-time and night-time temperatures in the areas being grazed may also affect grazing behaviour, albeit unfortunately this variable was not measured.

Another study conducted in central New Mexico (NM, USA) by Wesley et al. [13] studied the grazing behaviours of free-ranging beef cows. Based on their findings, two contrasting grazing personality types were described: type 1 cows, which used larger areas and displayed less twisted trajectories (21 ± 0.3 ha/d, 264 ± 8.9 m/d, respectively); and type 2 cows, which covered smaller areas and exhibited more tortuous trajectories (17 ± 2.0 ha/d, 314 ± 2.6 m/d, respectively). These results exemplified the home range versus movement tortuosity trade-off. It is noteworthy that the values reported by Wesley et al. [13] were closer to the values we recorded, than to those in the study of Browning et al. [32]. This may be because Wesley et al. [13] included all-of-day movement trajectories (as we did), instead of the grazing-time movement trajectories described by Browning et al. [32].

A similar analysis was also reported by Pauler et al. [48] for Swiss alpine pastures grazed with three different breeds of beef cattle. They observed that what are considered to be the more productive breeds (Braunvieh and Angus × Holstein cross cattle), took many more steps and covered longer distances but in much smaller areas, with this suggesting greater movement tortuosity. In contrast, the less productive Highland cattle appeared to explore larger areas but with fewer steps and shorter distance travelled, suggesting reduced movement tortuosity. While the grazing pattern of productive breeds suggested much greater grazing intensity (sensu Pauler et al. [48]) and therefore a high movement tortuosity, it also implied a much smaller portion of the accessible land was utilized, and probably high selectivity to graze a preferred and small area. The grazing pattern associated with the ‘less productive breed’ reflected individuals that explored a larger proportion of the available land, but with fewer steps. This suggests lower movement tortuosity and, as Pauler et al. [48] described, a much greater ‘evenness’ (i.e., on average all accessible vegetation patches were visited with similar frequency) denoting lower selectivity within the accessible land. The findings reported by Pauler et al. [48] suggest once again a trade-off between home range and movement tortuosity between breeds that displayed distinctive GP-behaviours.

Given the differences in experimental conditions between Browning et al. [32] and Wesley et al. [13], Pauler et al. [48] and the study here presented, it is notable that there are apparently similar trade-offs between home range and movement tortuosity. Furthermore, the genetic associations revealed here suggest that such trade-offs might be genetically controlled.

We acknowledge that some correlations between GP-behaviours might not have purely behavioural roots, but can instead be explained by other things such as through being mathematically related or being related through some environmental effect. For example, it is not surprising that the daily horizontal distance travelled and the daily three-dimensional distance travelled were highly correlated, because the former accounts for distance between two points in the same plane (a two-dimensional measure) and the latter accounts for changes in elevation as well as horizontal movement (a three-dimensional measure) by using the hypotenuse of the relocations. Equally, the vertical distance travelled is the sum of both elevation gains (ascent) and losses (descent), which while perhaps not equal, were nevertheless very similar in absolute values. Accordingly, the correlation of either with vertical distance travelled should be close to 100%. Additionally, in hill country rangelands steeper slopes occur at higher elevations, which is an environmental preconditioning for an animal grazing at higher elevation and steeper slopes as it is reflected with moderately positive correlations between the 85^th^ quantile of slope and relative elevation as well as between the daily slope 85^th^ quantile and the 85^th^ quantile of relative elevation. Overall, it could be concluded that not all correlations between GP-behaviours are necessarily meaningful from a behavioural viewpoint.

From an animal personality perspective, it is important to validate the correlation between grazing personality behaviours because this is a key premise to comply with the definition of animal personality [49]. Our results included correlation between several GP-behaviours measured on 303 individual cows over time (i.e., 7 to 28 d of recording GPS positions) and across contexts (i.e., different paddocks following the grazing rotation established by each farmer). Correlations among GP-behaviours have been reported in the past in beef cattle [13, 48, 50], dairy cattle [51–53], sheep [54, 55] and other domesticated livestock [56] and all support our findings. There is also evidence of the temporal consistency of such correlations in livestock [10, 57–59]. Changes in GP-behaviours have been also reported in dairy cattle [60], where they have been called personality developments and which are likely explained by regulating mechanisms such as animal maturity [59]. It might be concluded then that the behaviours investigated here comply with a definition of animal personality and might therefore be useful permanent descriptors of grazing personality for beef cattle and other foragers (see Moreno García et al. [12]). However, further investigation of behaviours that describe key traits of grazing personality (GP-traits) also seem to be warranted [49, 61, 62].

### Variation in the bovine *GRM5* gene

Investigation of the variation revealed in bovine *GRM5* exon 5 resulted in the discovery of three variant sequences, which were the result of two ‘silent’ nucleotide substitutions registered previously (rs43744222 and rs210610001). The presence of three synonymous single-nucleotide polymorphisms (SNPs) that do not change the amino acid coding in *GRM5* exon 5 does not mean these changes in nucleotide sequences are benign or innocuous, but rather they might lead to deleterious outcomes as exemplified with other genes [63]. Such silent substitutions might affect an animal’s functioning by a number of means that have been well described by Hurst [63]. For example, the nucleotide change might affect the rate of transcription and translation, and hence the folding of the peptide produced into a three-dimensional structure, which in turn may affect its function. Additionally the nucleotide changes may be linked to sequence variation elsewhere in the gene that has functional effect, or sequence variation in another closely linked gene that has a functional effect, given the linear arrangement of genes on chromosomes. Alternatively, nucleotide changes can affect the splicing and processing of the primary transcript, and thus modify the mRNA (and thus amino acid sequence produced at translation) or the regulation of translation. In the behavioural context, Fu et al. [64] illustrated that the effects of silent mutations on *Drosophila* circadian rhythm and thus, its potential implications on the regulation of animal daily and seasonal behaviours in general, which could apply to free-range cattle in steep and rugged terrain [65]. Considering the previously reported associations of *GRM5* with behaviour, movement and cognitive abilities in several animal species [15, 17–19, 21], the variants of bovine *GRM5* reported here are notable, because of their potential for use in cattle selection programmes that could target particular grazing patterns and cattle distribution in rangelands.

The proportion of genotypes was asymmetrical (Table 3) reflecting the low frequency of *GRM5* variant *A*. The *AA* genotype was only present in three cows from three different farms, and this did not allow any sensible comparison with others genotypes as any analysis is likely biased by the small sample-size. Moreover, as might be expected *AB* and *AC* were among the less common genotypes with frequencies of approximately 6% and 10%, respectively. These proportions were similar across the four farms, with this suggesting that in Hereford herds in New Zealand, natural or breeding-mediated factors had led to selection away from variant *A*.

Phenotypically, the *AB* and *AC* cows tended to explore larger areas in a slighter wider range of elevations, while displaying straighter trajectories (see Tables 5 and S2). From a genetic viewpoint, variant *A* was associated with elevation range, home range and movement tortuosity (Table 4); hence selection for more *A* in herds could result in changes to grazing patterns. It could then be further hypothesized that selecting for *A* at the expense of cattle with *B* (which had the largest daily movement tortuosity), would lead to differences in collective grazing personalities. Taking the above-mentioned example from Pauler et al. [48], selecting towards *A* could therefore increase the proportion of the ‘Highland cattle-like’ grazing pattern. However, to confirm the changes of grazing patterns for entire herds (i.e., collective grazing personality *sensu* Moreno García et al. [12]), the genetic associations and trends towards association with phenotypic behaviours reported here need to be further investigated in larger populations and with better balance introduced into the design to insure all the possible genotypes were evenly represented. It must also be acknowledged that other variants might be found as cattle of differing breed and larger herds are studied.

### Genotype-to-phenotype effects on grazing patterns

The most important results arising from this study are probably the discovery of genetic effects over consistently displayed grazing patterns in cattle and its potential for selecting individuals and designing herds based on desired grazing behaviours. The potential for genetic associations were investigated for simple behaviours (e.g., daily horizontal distance travelled, daily elevation gain and daily home range) as well as with a variable (daily movement tortuosity) that was constructed from the daily horizontal distance travelled and the daily home range. Associations were also investigated with so-called ‘relative’ behaviours, which express the grazing behaviour of an individual cow relative to the average behaviour of the herd. While associations were revealed with the simple GP-behaviours, no associations were found with relative behaviours. This latter approach attempts to fairly compare cows tracked under different conditions (e.g., on different farms and for different years), albeit the need to have an unbiased comparison was addressed in this study by correcting the mixed models with explanatory factors.

Interestingly, our results revealed trends for association and associations between *GRM5* variation and horizontal distance travelled and home range, respectively (Tables 4 and 5). Furthermore, we discovered stronger genetic association with daily movement tortuosity (Table 5), in part confirming the validity of the genetic effects on horizontal distance travelled and home range. Previous studies of *GRM5* genetic associations with indexes of terrain use in cattle have yielded contradicting results with Bailey et al. [15] describing associations, but Pierce et al. [16] failing to find associations. We hypothesize that one reason for the failure to detect genetic associations by Pierce et al. [16] could be their use of created or synthetic indexes that integrated two or more simple GP-behaviours, but in a normalized and averaged manner that rank individual cows according to the behaviours measured. This would be consistent with the lack of associations with the relative behaviours reported in the present study. It is unknown whether the simple behaviours chosen by Pierce et al. [16] (with or without normalization and centring) would have shown any signs towards genetic association. In this study, no association was revealed between *GRM5* variation and the 85^th^ quantile of daily slope, which is consistent with Pierce et al. [16]. Overall, it could be concluded that the reporting of trends toward genetic association (i.e., when *P* < 0.1) for simple variables is required to investigate and better understand potentially stronger associations with constructed variables that denote behavioural patterns, such as those observed in cattle grazing personality.

This study revealed *GRM5* exon 5 associations with daily home range and daily movement tortuosity (Table 5), which were age-dependent and that implied variation in grazing patterns among genotypes. Homozygous *BB* cows displayed the smallest daily home range and the most tortuous trajectories, while *AB* and *AC* cows had among the largest home ranges and straighter trajectories. Such observations applied well for four-year-old and older cows, but were not obvious for younger cows where *BB* individuals displayed similar home ranges and movement tortuosity to the *BC* and *CC* genotypes (Table 5). Behaviour changes during an animal’s lifetime are known as personality development and may occur for a variety of intrinsic reasons including genetic and epigenetic regulation, neurological and hormonal effects, among others [66] and may be affected by external reasons such as the social environment [12], for example. In dairy cattle, Neave et al. [59] studied the behavioural reactivity to novelty over the maturation of individuals from 1 month of age to 30 months. The experiment involved two longitudinal observations of 32 female Holstein cattle each, where individuals were evaluated with three personality tests (i.e., response to a novel environment, a novel human and a novel object), which were conducted on consecutive days at four defined times of development (i.e., pre-weaning, post-weaning, puberty and first lactation). Some behaviours were consistent between the pre-weaning and post-weaning stages, as well as between puberty and the first lactation; but the consistency was absent when behaviours were compared before and after cows’ sexual maturity (i.e., behaviours measured either in pre-weaning or post-weaning were inconsistent to measurements conducted later on over lactation). The authors concluded that personality traits became more consistent after sexual maturation and pointed out the need for studies beyond the first lactation [59]. Because the phenotypic expression of the *BB* genotype seems to be expressed in four-year-old and older cows in this study, it suggests that grazing personality is still developing in younger cattle, and supports the notion that an animal’s maturity affects grazing personality.

Regardless *GRM5* genotype, in our study, older cows displayed smaller home ranges and reached steeper terrain than younger cows (see section Cow age-class as main factor). This suggests that younger cows were able to graze larger areas displaying untwisted trajectories because they use gentler terrain, which contradicts the effects of cow age on the use of steep and rugged terrain previously reported [67, 68].

Are there potential opportunities in selecting cattle based on *GRM5* genotypes? With the information collected, one cannot be certain about the impact of selecting based on *GRM5* variation, but an estimate of the size of the effect can be ascertained from the differences in the marginal means in the GLMMs. Within a given cow age-class, differences in the marginal means for genotypes with the lowest and highest values of home range and movement tortuosity were 19% and 23%, respectively (section Cow age-class as main factor). For home range, the *BB* cows had the lowest marginal mean while the highest was for *AB* cows. Inversely, the lowest marginal mean of movement tortuosity was modelled for the *AB* genotype and the highest for *BB* (i.e., same genotypes but in opposite ends), which is supported by the negative correlation between the two GP-behaviours. We speculate that such differences could be higher if cows with genotype *AA* were well represented in cattle herds and therefore could be included in the comparisons. Even with these results, a change of roughly 20% in daily home range and approximately 23% of movement tortuosity over the explored area could make a notable difference in rangeland use, ecological functioning and eventually in cattle production. Further research is encouraged to elucidate the benefits of applying grazing personality in cattle selection programmes.

## Conclusion

Our study revealed the association of glutamate metabotropic receptor 5 gene (*GRM5*) variation with home range and movement tortuosity that could possibly be used in cattle breeding programmes to improve rangeland utilisation and grazing distribution. There appeared to be a genetically determined trade-off between the daily home range and daily movement tortuosity. Our research also showed a widespread association between cow age-classes and most behaviours of grazing personality with two interesting implications. Firstly, grazing personality development occurs beyond a cow reaching her sexual maturity and it appears to stabilise in 4-year-old cows. Secondly, cows of younger age classes grazed larger but gentler areas, while displayed straighter trajectories than their counterparts older cows. In this study, three novel sequence variants of *GRM5* exon 5 were revealed, and these had different frequencies in the Hereford cattle. The asymmetric occurrence of *GRM5* variation offers the opportunity to shape the grazing patterns of beef cattle through selection.

## Supplementary Information

**Additional file 1: Fig. S1.** Residuals versus fitted values (Part A). Plots of scaled residuals versus fitted values of linear mixed models of six (A-F) grazing personality behaviours. Residual outliers are values beyond ±3.

**Additional file 2: Fig. S2.** Residuals versus fitted values (Part B). Plots of scaled residuals versus fitted values of linear mixed models of five (A-E) grazing personality behaviours. Residual outliers are values beyond ±3.

**Additional file 3: Table S1.** Akaike Information Criterion (AIC) for linear mixed models of grazing personality behaviours (GP-behaviours) fitted with twelve combinations of random factors.

**Additional file 4: Table S2.** Associations between genotypes of the glutamate metabotropic receptor 5 gene (*GRM5*) and grazing personality behaviours (GP-behaviours).

## Data Availability

The GPS data that support the findings of this study were deposited in Movebank with the identifiers Movebank ID 1321429570 and Movebank ID 1321461925 and available from the corresponding author on reasonable request.

## Abbreviations

AIC: Akaike information criterion
BIC: Bayesian information criterion
DEM: Digital elevation model
GLMM: Generalised linear mixed model(s)
GP-behaviours: Grazing personality behaviour(s)
GP-model: Model of grazing personalities
GPS: Global positioning system
GRMs: Metabotropic glutamate receptors
GRM1: Metabotropic glutamate receptor 1 (G-protein)
GRM5: Metabotropic glutamate receptor 5 (G-protein)
*GRM5*: Glutamate metabotropic receptor 5 gene
LMM: Linear mixed model(s)
PCR: Polymerase chain reaction
QTL: Quantitative trait loci
SSCP: Single strand conformation polymorphism
USA: United states of America
bp: Base pairs
d: Day(s)
ha/d: Hectares per day
m: Metres
m/ha: Metres per hectare
s: Second(s)
h: Hour(s)
min: Minute(s)
V: Volts
°: Angular degrees
°C: Celsius degrees
ele_gain: Daily elevation gain
ele_range: Daily elevation range
ho_dist: Daily horizontal distance travelled
hr_mcp: Daily home range
rel_ele: Relative elevation mean
rel_ele_range: Relative elevation range
rel_ele85: Relative 85^th^ quantile of elevation
slope85: 85^th^ quantile of daily slope
sp_tortuosity: Daily movement tortuosity
ve_dist: Daily vertical distance travelled
3D_dist: Daily three-dimensional distance travelled
