# Supplementary material for: The glutamate metabotropic receptor 5 (GRM5) gene is associated with beef cattle home range and movement tortuosity
Source: J Anim Sci Biotechnol. 2022 Sep 15;13:111. doi: 10.1186/s40104-022-00755-7 (PMC9476267; doi:10.1186/s40104-022-00755-7)
Supplement: Supplementary file 1 — Additional file 1: Fig. S1. Residuals versus fitted values (Part A). Plots of scaled residuals versus fitted values of linear mixed models of six (A-F) grazing personality behaviours. Residual outliers are values beyond ±3. [file 40104_2022_755_MOESM1_ESM.docx]

Fig. S1. Residuals versus fitted values (Part A). Plots of scaled residuals versus fitted values of linear mixed models of six (A-F) grazing personality behaviours. Residual outliers are values beyond ±3.


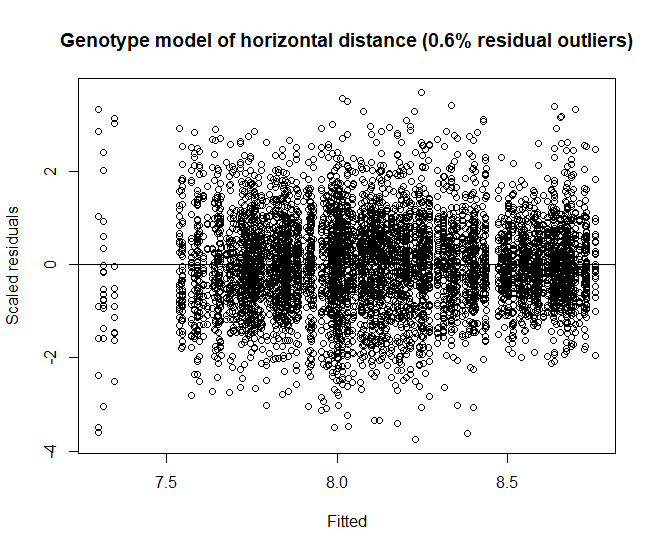

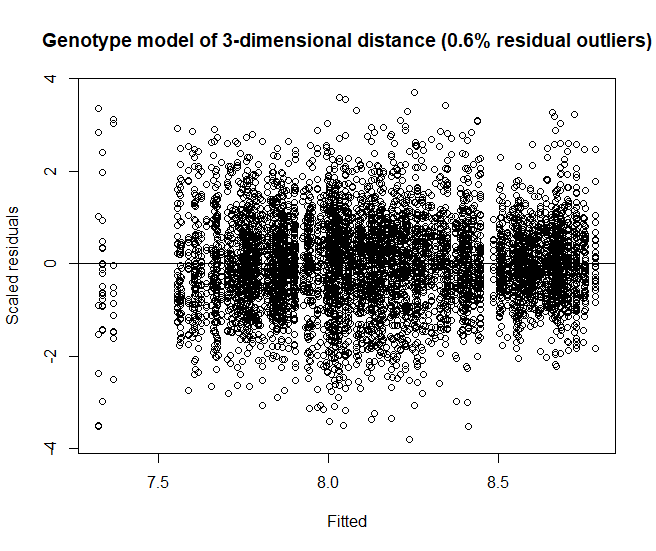

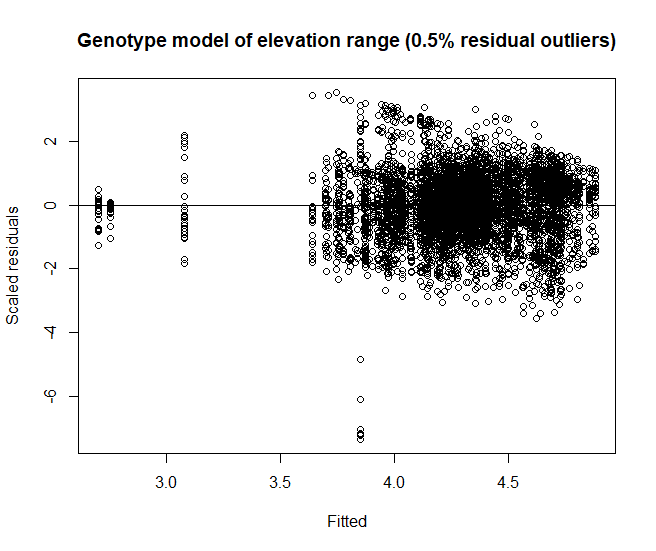

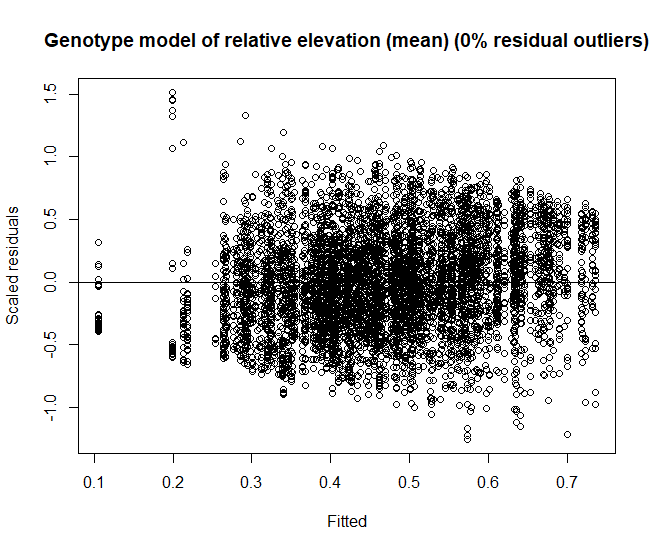

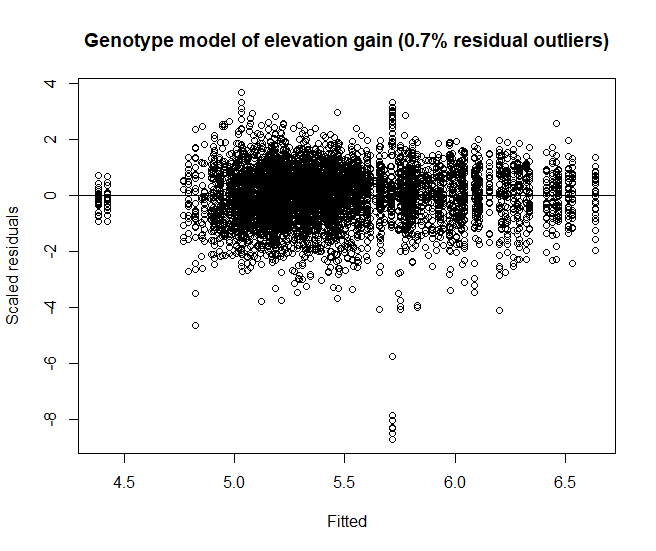

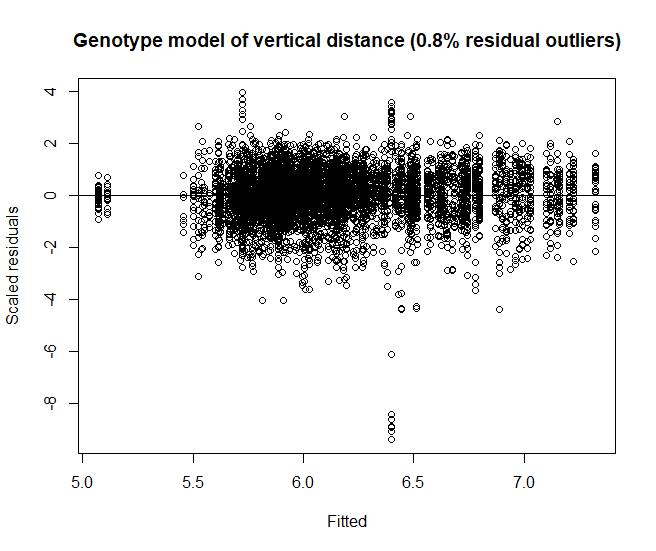


A

B

C

D

E

F
