# Supplementary material for: The glutamate metabotropic receptor 5 (GRM5) gene is associated with beef cattle home range and movement tortuosity
Source: J Anim Sci Biotechnol. 2022 Sep 15;13:111. doi: 10.1186/s40104-022-00755-7 (PMC9476267; doi:10.1186/s40104-022-00755-7)
Supplement: Supplementary file 2 — Additional file 2: Fig. S2. Residuals versus fitted values (Part B). Plots of scaled residuals versus fitted values of linear mixed models of five (A-E) grazing personality behaviours. Residual outliers are values beyond ±3. [file 40104_2022_755_MOESM2_ESM.docx]

Fig. S2. Residuals versus fitted values (Part B). Plots of scaled residuals versus fitted values of linear mixed models of five (A-E) grazing personality behaviours. Residual outliers are values beyond ±3.


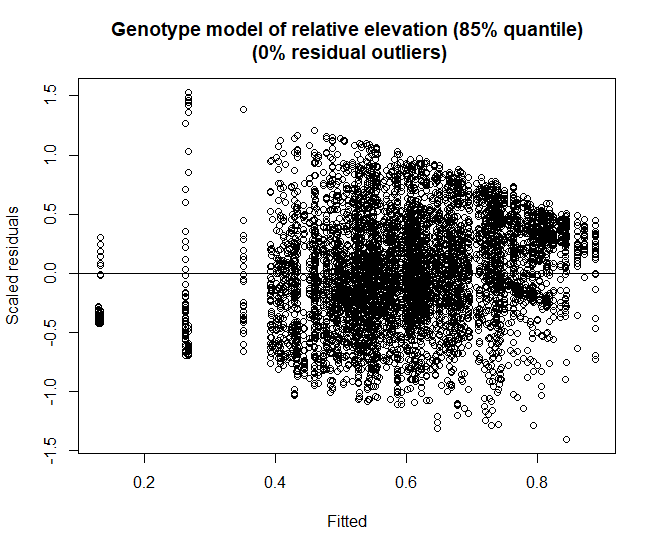

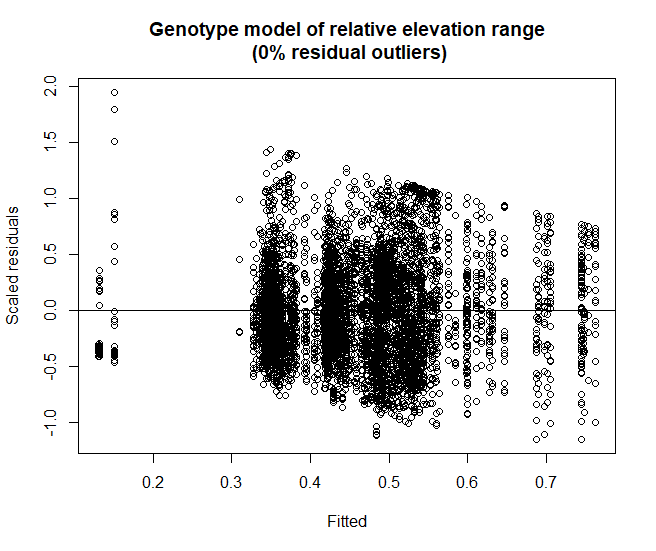

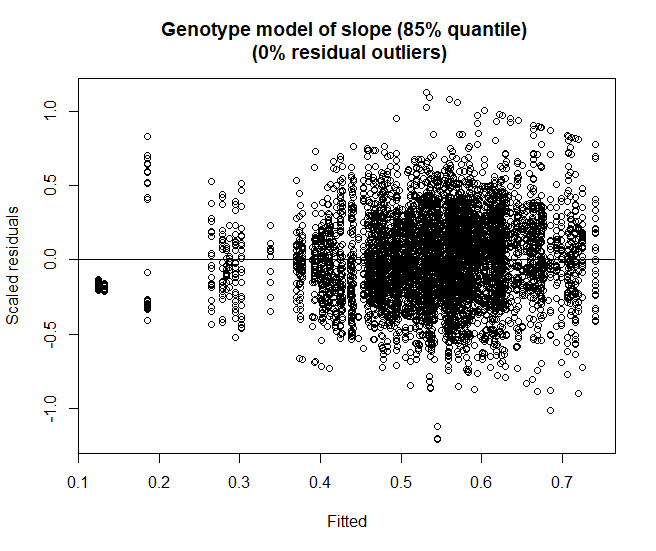

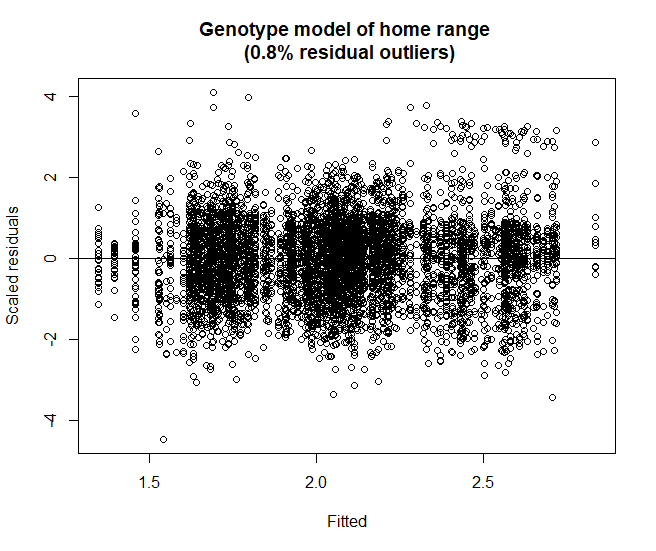

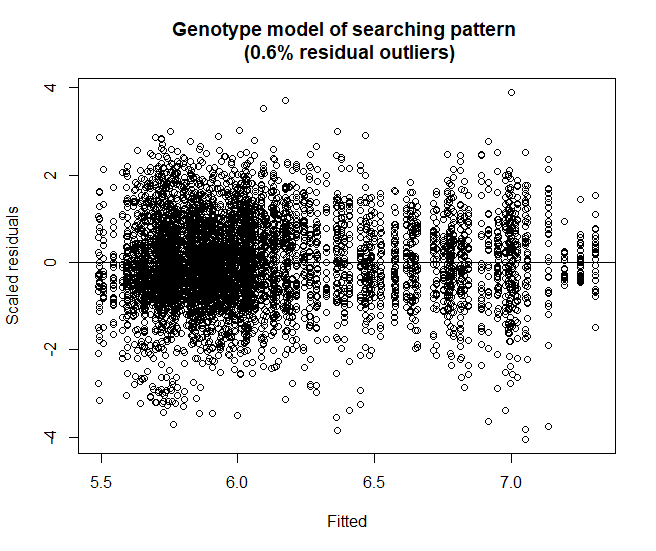


**Genotype model of spatial search pattern (0.6% residual outliers)**

A

B

C

D

E
