# Supplementary material for: The glutamate metabotropic receptor 5 (GRM5) gene is associated with beef cattle home range and movement tortuosity
Source: J Anim Sci Biotechnol. 2022 Sep 15;13:111. doi: 10.1186/s40104-022-00755-7 (PMC9476267; doi:10.1186/s40104-022-00755-7)
Supplement: Supplementary file 3 — Additional file 3: Table S1. Akaike Information Criterion (AIC) for linear mixed models of grazing personality behaviours (GP-behaviours) fitted with twelve combinations of random factors. [file 40104_2022_755_MOESM3_ESM.docx]

Table S1. Akaike Information Criterion (AIC) for linear mixed models of grazing personality behaviours (GP-behaviours) fitted with twelve combinations of random factors

| **Random Factors**^1^ | **Degrees of freedom** | | **ho_dist^3^** | **ve_dist** | **3D_dist** | **ele_ran-ge** | **ele_gain** | **Rel_ele** | **rel_ele-85** | **rel_ele_range** | **slope85** | **hr_mcp** | **sp_tortuosity** |
| --- | --- | --- | --- | --- | --- | --- | --- | --- | --- | --- | --- | --- | --- |
|  | **(Ga)^2^ (Bi)^2^** | | **(Ga)** | **(Ga)** | **(Ga)** | **(Ga)** | **(Ga)** | **(Bi)** | **(Bi)** | **(Bi)** | **(Bi)** | **(Ga)** | **(Ga)** |
| (1\|cow_id) | 3 | 2 | 898 | 5051 | 797 | 9820 | 5880 | 7815 | 7359 | 7915 | 7906 | 15781 | 13861 |
|  |  |  |  |  |  |  |  |  |  |  |  |  |  |
| (1\|cow_id)+ (1\|farm_id) | 4 | 3 | 816 | 5050 | 717 | 9703 | 5879 | 7728 | 7269 | 7892 | 7881 | 15719 | 13863 |
|  |  |  |  |  |  |  |  |  |  |  |  |  |  |
| (1\|cow_id)+ (1\|sampling_year)+ (1\|farm_id) | 5 | 4 | 509 | 4892 | 405 | 9705 | 5723 | 7730 | 7270 | 7889 | 7883 | 15697 | 13597 |
|  |  |  |  |  |  |  |  |  |  |  |  |  |  |
| (1\|cow_id)+ (1\|sampling_year/farm_id) | 5 | 4 | **465^4^** | 4878 | **366** | 9693 | 5709 | 7727 | 7273 | 7802 | 7833 | 15597 | 13538 |
|  |  |  |  |  |  |  |  |  |  |  |  |  |  |
| (1\|cow_id)+ (1\|farm_id/sampling_year) | 5 | 4 | 470 | 4886 | 371 | 9693 | 5717 | 7726 | 7270 | 7802 | 7833 | 15597 | 13546 |
|  |  |  |  |  |  |  |  |  |  |  |  |  |  |
| (1\|cow_id)+ (1\|mob_id) | 4 | 3 | 478 | **4757** | 380 | 9538 | 5591 | **7717** | **7265** | 7704 | **7724** | 15573 | **13527** |
|  |  |  |  |  |  |  |  |  |  |  |  |  |  |
| (1\|cow_id) + (1\|sire_id) | 4 | 3 | 692 | 5006 | 593 | 9771 | 5836 | 7777 | 7322 | 7898 | 7903 | 15695 | 13795 |
|  |  |  |  |  |  |  |  |  |  |  |  |  |  |
| (1\|cow_id) + (1\|mob_id) + (1\|sire_id) | 5 | 4 | 479 | 4758 | 381 | **9540** | **5592** | 7718 | 7267 | **7700** | 7726 | **15570** | 13529 |
|  |  |  |  |  |  |  |  |  |  |  |  |  |  |
| (1\|cow_id) + (1\|mob_id) + (1\|sire_id) + (1\|GRM5_genotype) | 6 | 5 | 481 | 4760 | 383 | 9542 | 5594 | 7720 | 7269 | 7702 | 7728 | 15571 | 13527 |
|  |  |  |  |  |  |  |  |  |  |  |  |  |  |
| (1\|cow_id) + (1\|farm_id) + (1\|sampling_year) + (1\|GRM5_genotype) | 6 | 5 | 511 | 4894 | 407 | 9706 | 5724 | 7732 | 7272 | 7891 | 7885 | 15699 | 13599 |
|  |  |  |  |  |  |  |  |  |  |  |  |  |  |
| (1\|cow_id) + (1\|sampling_year/farm_id) + (1\|GRM5_genotype) | 6 | 5 | 467 | 4880 | 368 | 9695 | 5711 | 7729 | 7275 | 7804 | 7835 | 15595 | 13535 |
|  |  |  |  |  |  |  |  |  |  |  |  |  |  |
| (1\|cow_id) + (1\|sampling_year/farm_id) + (1\|sire_id) | 6 | 5 | 465 | 4878 | 366 | 9690 | 5709 | 7727 | 7727 | 7800 | 7835 | 15585 | 13535 |

^1^Random factors presented with the notation of the ’lme4r’ R package for cow identity (cow_id, *n* = 303), farm (farm_id, *n* = 4), year of sampling (sampling_year, *n* = 2), herd per farm and per year (mob_id, *n* = 14), sire identity (sire_id, *n* = 73), *GRM5* genotype (GRM5_genotype, *n* = 5).

^2^Models set with Gaussian (Ga) or Binomial (Bi) distribution according to the distribution of each variable.

^3^See GP-behaviours abbreviations and details in Table 1.

^4^Bolded and underlined AIC values indicate the combination of random factors selected to fit models with fixed factors
