# Supplementary material for: The glutamate metabotropic receptor 5 (GRM5) gene is associated with beef cattle home range and movement tortuosity
Source: J Anim Sci Biotechnol. 2022 Sep 15;13:111. doi: 10.1186/s40104-022-00755-7 (PMC9476267; doi:10.1186/s40104-022-00755-7)
Supplement: Supplementary file 4 — Additional file 4: Table S2. Associations between genotypes of the glutamate metabotropic receptor 5 gene (GRM5) and grazing personality behaviours (GP-behaviours). [file 40104_2022_755_MOESM4_ESM.docx]

Table S2. Associations between bovine *GRM5* genotypes and grazing personality behaviours (GP-behaviours)

| **GP-behaviour**^1^ | **Cow**  **age-class**  **(*P*-value)**^2^ | **Geno-type** | **Marginal mean**^3^  **(standard error)** | | ***P*-value**^4^ |
| --- | --- | --- | --- | --- | --- |
| ho_dist, m/d | **0.043** | *AB* | 3655 | (±1071) | _^4^ |
|  |  | *AC* | 3950 | (±1154) | *0.085* |
|  |  | *BB* | 3818 | (±1115) | 0.319 |
|  |  | *BC* | 3885 | (±1131) | 0.117 |
|  |  | *CC* | 3842 | (±1119) | 0.199 |
|  |  |  |  |  |  |
| ve_dist, m/d | 0.692 | *AB* | 540 | (±75) | _ |
|  |  | *AC* | 583 | (±79) | 0.265 |
|  |  | *BB* | 558 | (±75) | 0.614 |
|  |  | *BC* | 561 | (±74) | 0.527 |
|  |  | *CC* | 542 | (±72) | 0.952 |
|  |  |  |  |  |  |
| 3D_dist, m/d | **0.049** | *AB* | 3713 | (±1089) | _ |
|  |  | *AC* | 4015 | (±1174) | *0.084* |
|  |  | *BB* | 3881 | (±1134) | 0.316 |
|  |  | *BC* | 3946 | (±1150) | 0.120 |
|  |  | *CC* | 3901 | (±1137) | 0.204 |
|  |  |  |  |  |  |
| ele_range, m | 0.427 | *AB* | 72 | (±9) | _ |
|  |  | *AC* | 71 | (±9) | 0.797 |
|  |  | *BB* | 65 | (±8) | *0.070* |
|  |  | *BC* | 66 | (±8) | *0.095* |
|  |  | *CC* | 65 | (±8) | *0.064* |
|  |  |  |  |  |  |
| ele_gain, m/d | 0.699 | *AB* | 272 | (±38) | _ |
|  |  | *AC* | 291 | (±39) | 0.336 |
|  |  | *BB* | 280 | (±38) | 0.645 |
|  |  | *BC* | 281 | (±37) | 0.593 |
|  |  | *CC* | 270 | (±35) | 0.889 |
|  |  |  |  |  |  |
| rel_ele  (0-1) | **0.017** | *AB* | 0.40 | (±0.07) | _ |
|  |  | *AC* | 0.43 | (±0.06) | 0.606 |
|  |  | *BB* | 0.46 | (±0.06) | 0.316 |
|  |  | *BC* | 0.44 | (±0.06) | 0.426 |
|  |  | *CC* | 0.48 | (±0.06) | 0.132 |
|  |  |  |  |  |  |
| rel_ele85  (0-1) | **0.006** | *AB* | 0.66 | (±0.07) | _ |
|  |  | *AC* | 0.68 | (±0.06) | 0.725 |
|  |  | *BB* | 0.67 | (±0.06) | 0.835 |
|  |  | *BC* | 0.66 | (±0.05) | 0.978 |
|  |  | *CC* | 0.69 | (±0.05) | 0.527 |
|  |  |  |  |  |  |
| rel_ele_range  (0-1) | 0.280 | *AB* | 0.50 | (±0.09) | _ |
|  |  | *AC* | 0.50 | (±0.09) | 0.854 |
|  |  | *BB* | 0.48 | (±0.09) | 0.649 |
|  |  | *BC* | 0.48 | (±0.08) | 0.650 |
|  |  | *CC* | 0.48 | (±0.08) | 0.747 |
|  |  |  |  |  |  |
| slope85  (0-1) | **0.020** | *AB* | 0.44 | (±0.09) | _ |
|  |  | *AC* | 0.49 | (±0.09) | 0.323 |
|  |  | *BB* | 0.48 | (±0.09) | 0.412 |
|  |  | *BC* | 0.43 | (±0.08) | 0.718 |
|  |  | *CC* | 0.43 | (±0.08) | 0.764 |
|  |  |  |  |  |  |
| hr_mcp, ha/d | **0.008** | *AB* | 7.88 | (±0.91) | _ |
|  |  | *AC* | 7.82 | (±0.86) | 0.917 |
|  |  | *BB* | 6.6 | (±0.73) | **0.015** |
|  |  | *BC* | 7.25 | (±0.75) | 0.189 |
|  |  | *CC* | 7.29 | (±0.76) | 0.223 |
|  |  |  |  |  |  |
| sp_tortuosity, m/ha | **0.008** | *AB* | 540 | (±75) | _ |
|  |  | *AC* | 568 | (±77) | 0.436 |
|  |  | *BB* | 665 | (±90) | **0.001** |
|  |  | *BC* | 616 | (±81) | **0.019** |
|  |  | *CC* | 602 | (±80) | *0.053* |
| ^1^See GP-behaviours abbreviations and details in Table 1.  ^2^Significance level of ANOVA tests for comparison of models with and without cow age-class as a fixed factor. Bold letters indicate significance (*P <* 0.05).  ^3^Marginal mean in measured units (back-transformed from the log scale as needed) as estimated with linear mixed models.  ^4^Significance level for Satterthwaite’s method *t*-tests. Italicised values indicate trend to significance (*P* *<* 0.1) and bolded values indicate significance (*P* *<* 0.05).  ^5^Model intercepts set with genotype *AB* and cow age-class 1 | | | | | |
